# Doxorubicin-induced cardiometabolic disturbances: what can we do?

**DOI:** 10.3389/fcdhc.2025.1537699

**Published:** 2025-02-14

**Authors:** Ashot Avagimyan, Nana Pogosova, Manfredi Rizzo, Nizal Sarrafzadegan

**Affiliations:** ^1^ Yerevan State Medical University after M. Heratsi, Yerevan, Armenia; ^2^ Isfahan Cardiovascular Research Centre, Cardiovascular Research Institute, Isfahan University of Medical Sciences, Isfahan, Iran; ^3^ Sciences and Preventive Cardiology, National Medical Research Centre of Cardiology after Acad. E. I. Chazov, Moscow, Russia; ^4^ Evidence-Based Medicine Department, Institute of Medicine, Patrice Lumumba Peoples' Friendship University of Russia (RUDN), Moscow, Russia; ^5^ Cardiometabolic Medicine Department, University of Palermo, Palermo, Italy

**Keywords:** doxorubicin, chemotherapy, metabolism, cardiometabolic, myocardium, glucose, GLUT, insulin resistance

Metabolic syndrome represents a significant contemporary crisis on a global scale, creating a conducive ground for cardiovascular diseases (CVDs) and cancer development; in this context, the exploration of potential therapies aimed at correcting metabolic dysfunctions in the realm of preventive cardio-oncology appears to be particularly promising (1–3).

Cardio-oncology is an emerging specialty within the field of internal medicine. Despite an extensive history of research in this area, we face more questions than answers, as highlighted by the first consensus guidelines provided by the European Society of Cardiology (4), in which only 3% of the guidelines hold an evidence level of “A.” Furthermore, the proposed algorithms for primary and secondary prevention primarily focus on suppressing neurohumoral activation, advocating for a combination of angiotensin-converting enzyme (ACE) inhibition or angiotensin receptor blockade (ARB) alongside β-blockers. There is also an ongoing discussion regarding the rationale behind prescribing statins and mineralocorticoid inhibitors.

The therapeutic landscape in cardiology shows that nearly all available treatment options are being utilized in cardio-oncology. It is noteworthy that, according to analyses of real clinical practice, the established combination of ACEi/ARB with β-blockers is linked to a 42% responders’ rate; conversely, non-responders in this group experience a decline in the clinical status among cardio-oncological patients (5). This observation, alongside the challenges of prescribing this combination to normotensive cancer patients, raises questions about the true clinical effectiveness of this strategy, particularly in the fashion of statistically insignificant changes in heart failure incidence (6).

Additionally, the only specific cardioprotective agent currently recognized is dexrazoxane, an iron chelator that modifies doxorubicin (DOX)-associated ferroptosis and oxidative damage in cardiomyocytes. Though its use is limited, and the drug has sparked considerable debate regarding its potential negative impact on oncological outcomes, it is worth noting that an increasing number of randomized clinical trials and meta-analyses are emerging that indicate no adverse effects of this medication on cancer prognosis, but this question is still open and debatable (7–9).

What accounts for the “failure” of studies conducted in cardio-oncology over the past seven decades? Two primary factors stand out: first, most foundational studies were performed predominantly on initially healthy male experimental subjects, utilizing a single chemotherapy drug without tumor modeling and with a wide spectra of experimental models; that is why the proposed studies have a significant number of limitations, which make it hard to compare. Second, all cardioprotective compounds investigated rely, one way or another, on the antioxidant AKT/PI3K/Sirt3/Nrf2 cascade activation. However, translating these findings into clinical practice has shown no clinically significant effects. Traditionally, oxidative stress has been viewed as the initiating factor in the pathogenesis of cancer therapy-related cardiovascular toxicity (CTR- CVT) and cancer therapy-related cardiac dysfunction (CTRCD) (10). However, the modern scenario indicates that DOX-based chemotherapy is associated with a range of cardiometabolic disorders (11–15):

1. DOX treatment correlates with elevated glucose levels. Hyperglycemia, insulin resistance, and heightened free fatty acid levels emerge in both acute and chronic models of cardiotoxicity, typically due to disturbances in glucose metabolism within striated muscles. As part of our clinical investigation into the issues of hyperglycemia, prediabetes, and type 2 diabetes mellitus (T2DM) in cardio-oncology, our research team is examining metabolic disorders in the Cardiovascular Events in Breast and Colorectal Cancer (CIBC) cohort, headed by Prof. Nizal Sarrafzadegan, focusing on the effects of chemotherapy on cancer patients with or without T2DM.
2. Furthermore, treatment with DOX is associated with reduced activity of GLUT-1 and GLUT-4, a dose- and time-dependent decline in PPARγ expression, and an increase in miR-130a levels. DOX-based chemotherapy leads to glucose metabolism disturbances not only in the endothelium but also in the myocardium. Additionally, DOX provides versatile toxic impairment on pancreatic B cells.
3. Despite an overall reduction in body weight, DOX treatment leads to the development of atherogenic dyslipidemia with augmented serum triglyceride levels, characterized by elevated non-HDL cholesterol, a high triglyceride/glucose index, and increased atherogenicity.

Upon analyzing the information above, it is important to highlight the increasing interest in the study of relatively new antidiabetic drugs within the field of cardio-oncology, particularly sodium glucose co-transporter-2 inhibitors (SGLT2i) such as dapagliflozin and empagliflozin, and glucagon-like peptide-1 (GLP-1) receptor agonists like semaglutide and tirzepatide. While the emerging evidence underscores the substantial cardioprotective potential of semaglutide and tirzepatide in cardiology, their widespread use among cancer patients presents significant challenges due to their pancreatotoxic effects and the frequent occurrence of severe gastrointestinal complications.

In this context, SGLT2i appears especially relevant. Current research indicates that gliflozins, in addition to their primary metaboprotective effects, possess a range of pleiotropic mechanisms, such as antioxidant, energy stabilization, endothelium protection, and anti-inflammatory effects, that not only positively influence the heart, kidneys, nervous system, and liver but also serve some anti-cancer properties (16–18).

The advantages of SGLT2i, coupled with their endorsement as an initial therapeutic agent for heart failure beyond the entire spectrum of left ventricular ejection fraction, underscore the relevance of assessing SGLT2i in cardio-oncology. In light of this, our research group aims to not only evaluate the incidence of chemotherapy-related glucose metabolism disturbances at the population level (CIBC cohort); we also aimed to investigate the rationale for dapagliflozin prescription to prevent metabolic disorders and cardiovascular complications of chemotherapy-related cardiotoxicity. Our preclinical studies will be conducted on our experimental model (Avagimyan et al. (19)) in female rats with 7,12-dimethylbenz[a]anthracene (DMBA)-induced breast cancer. Preliminary evaluation suggests that dapagliflozin enhances the structural and functional parameters of CVD without any pathomorphological (immunohistochemical) data exacerbating the oncological process.

In analyzing these findings, it is essential to emphasize the need for future assessments of chemotherapy-related metabolic disturbances. This should involve the integration of cardiometabolic screening strategies into clinical practice for both primary and secondary prevention of cardiovascular events, along with cardiac rehabilitation for cancer patients. Therefore, further investigation into the effects of SGLT2i for the cardiometabolic toxicity prevention in undertreated and post-treatment cancer patients is needed.
